# Antimicrobial Intervention by Photoirradiation of Grape Pomace Extracts via Hydroxyl Radical Generation

**DOI:** 10.3389/fphys.2017.00728

**Published:** 2017-09-21

**Authors:** Yoshimi Niwano, Mika Tada, Mana Tsukada

**Affiliations:** ^1^Graduate School of Dentistry, Tohoku University Sendai, Japan; ^2^Center for General Education, Tohoku Institute of Technology Sendai, Japan; ^3^HABA Laboratories Inc. Tokyo, Japan

**Keywords:** grape pomace, photooxidation of polyphenol, hydroxyl radical, electron spin resonance, spin trapping

## Abstract

The annual production of grape worldwide amounts to almost 70 million tons, and around 80% is used for winemaking. The two major wastes from winemaking process, pomace and lees account for 20 and 7% of the grapes, respectively. They have been expected as a valuable resource to be recycled because they are rich in polyphenols. Polyphenols possess prooxidatve activity as well as antioxidative one just like a two sides of a coin. A typical example of the prooxidative activity is antibacterial activity of catechins. The activity is exerted through oxidation of phenolic hydroxyl moiety coulpled with reduction of dissolved oxygen leading to hydrogen peroxide (H_2_O_2_) generation. In addition, once the oxidation of phenolic hydroxyl moiety is augmented by photoirradiation, highly reactive hydroxyl radical (·OH) is generated. Accordingly, there have been several reports showing that photoirardiation of polyphenols exerts bactericidal activity via ·OH generation. This review focuses mainly on antimicrobial intervention by photoirradiation of grape pomace extract in relation to ·OH generation analyzed by an electron spin resonance-spin trapping method.

## Prooxidative property of polyphenols

Polyphenolic compounds naturally occurring in fruits, nuts, vegetables and flowers are noteworthy for their antioxidative activity (Kondo et al., 1999; Liu et al., 2000; Yilmaz and Toledo, 2004). The phenolic hydroxyl moiety in their structures functions as a hydrogen (and electron) donor, which enables them to scavenge free radicals effectively (Hanasaki et al., 1994; Heim et al., 2002). Accordingly, polyphenols in terms of their beneficial antioxidative activity have been well studied and applied to health promotion (Ahmad et al., 2000; Williamson and Manach, 2005; Khan and Mukhtar, 2007).

In addition to antioxidative potential of polyphenols, their prooxidative potential has been also studied. One of the typical examples is tea catechin with cupric ion showing a prooxidative activity to DNA cleavage reaction and linoleic acid peroxidation (Hayakawa et al., 1997). More in detail, the catechin probably acts to reduce Cu^2+^ to Cu^+^ expressed as Equation i. Then the resultant Cu^+^ produces reactive oxygen species (ROS) attacking the DNA (Equations ii–iv). In the equations, superoxide anion radical is abbreviated as ·${\text{O}}_{2}^{-}$. Especially, the Eq iv is well known as a Fenton reaction in which transition metal catalyzes hydrogen peroxide (H_2_O_2_) to generate highly reactive hydroxyl radical (·OH).

(i) $$\begin{array}{r} \text{catechin}+\text{C}{\text{u}}^{2+}\to \text{oxidized catechin}+\text{C}{\text{u}}^{+} \end{array}$$

(ii) $$\begin{array}{r} \text{C}{\text{u}}^{+}+{\text{O}}_{2}\to \text{C}{\text{u}}^{2+}+\cdot {\text{O}}_{2}^{-} \end{array}$$

(iii) $$\begin{array}{r} 2\text{C}{\text{u}}^{+}+{\text{O}}_{2}+2{\text{H}}^{+}\to 2\text{C}{\text{u}}^{2+}+{\text{H}}_{2}{\text{O}}_{2} \end{array}$$

(iv) $$\begin{array}{r} \text{C}{\text{u}}^{+}+{\text{H}}_{2}{\text{O}}_{2}\to \text{C}{\text{u}}^{2+}+\cdot \text{OH}+\text{O}{\text{H}}^{-} \end{array}$$

Likewise, it was shown that an anticancer action of plant polyphenols is executed by intracellular copper mobilization and ROS generation, which would be a feature of prooxidative properties of polyphenols, leading to cancer cell death (Khan et al., 2014). More in detail, polyphenols including luteolin, apigenin, epigallocatechin-3-gallate, and resveratrol inhibited cell proliferation and induced apoptosis in different cancer cell lines, and such cell death was prevented by cuprous chelator neocuproine and ROS scavengers. Furthermore, normal breast epithelial cells cultured in a medium supplemented with copper could be suffer from such polyphenol-induced growth inhibition. As such, the high concentration of copper in cancer cells would be a crucial factor for the preferential cytotoxicity of several polyphenols with diverse chemical structures toward cancer cells.

**Figure 1:**
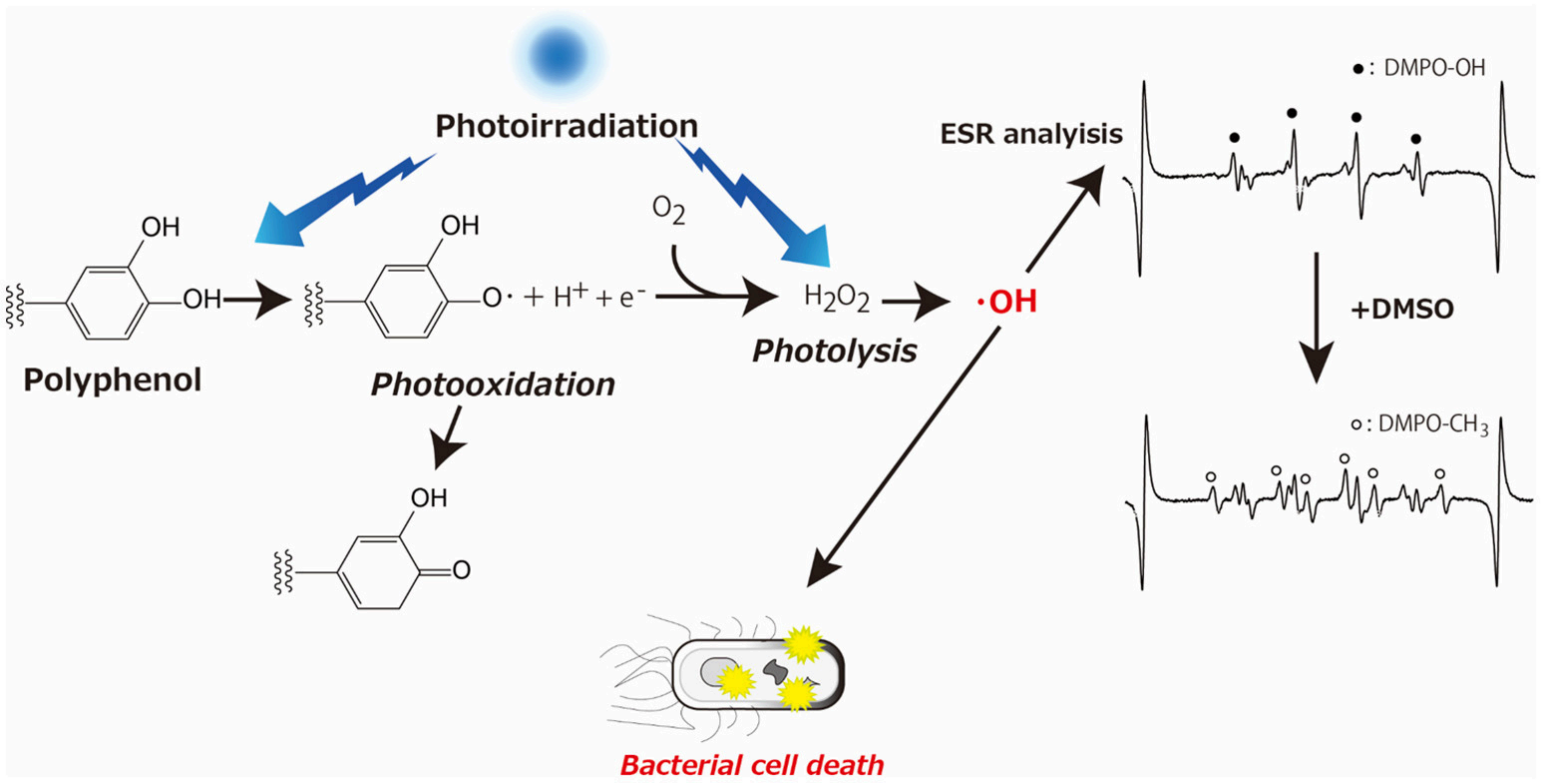
Schematic illustration showing augmented oxidation of phenolic hydroxyl moiety by photoirradiation, which results in hydroxyl radical generation leading to bacterial cell death, with representative ESR spectra obtained by photoirradiation of GPE in the absence or presence of DMSO.

The other typical example of prooxidative action of polyphenols is antibacterial activity of catechins. It was reported that catechins (epicatechin, epicatechin gallate, epigallocatechin and epigallocatechin gallate) possess strong bactericidal action due to ROS such as H_2_O_2_ generated through the oxidation of catechins as the active mechanism (Arakawa et al., 2004). Focusing attention on the antibacterial action of catechins, several studies have been conducted. These studies discuss augmented bactericidal action of polyphenols upon photoirradiation (Nakamura et al., 2012, 2013, 2015). To be more precise, photoirradiation of polyphenol aqueous solution led to the generation of H_2_O_2_ that was in turn homolytically cleaved to ·OH. The resultant ·OH caused oxidative damage leading to bacterial death. As illustrated in Figure 1, the concept of these studies is that photoirradiation could augment the oxidation of polyphenols, especially the phenolic hydroxyl moieties.

## Grape pomace as a natural resource of polyphenols

According to FAO (Food and Agriculture Organization) statistics, grape is the largest fruit crop in the world, and the annual production worldwide amounts to almost 70 million tons, and around 80% is used to make wine. The waste from winemaking process mainly consists of pomace, clarification sediment such as lees, and yeast sediment. Although the generated amount of waste depends on the condition of the grapes at the time of harvest, as well as on what processing methods are used, the levels of waste can be up to 20% of the harvested mass of the grapes (Russ and Meyer-Pittroff, 2004). Accordingly, such waste materials or byproducts could be a valuable resource to be recycled. Indeed, solid byproducts from white and red wine industry were evaluated as potential sources of antioxidant phytochemicals on the basis of their content in phenolic compounds and *in vitro* antioxidative activity (Markis et al., 2007). In particular, it is shown that wine industry byproducts, including not only grape seeds but also grape pomace and stems, were very rich sources of antioxidant polyphenols compared with other agri-food solid wastes. Regarding the phenolic compounds contained in winery waste, it was reported that gallic acid, (+)-catechin and (−)-epicatechin were the major phenolic compounds in the waste from red winemaking with variety Agiorgitiko, according to a high performance liquid chromatography determination of the extracts obtained under various conditions using different solvents (Lafka et al., 2007). Moreover, hydroxytyrosol, tyrosol, cyanidin glycosides, and various phenolic acids such as caffeic, syringic, vanillic, p-coumaric and o-coumaric acids were identified.

## ROS generation from photoirradiation of grape pomace extract

As described above, photoirradiation of polyphenols could exert bactericidal action via generated ROS, especially ·OH. In this review, we write up our recent studies showing the photoirradiation-induced bactericidal activity of an aqueous extract from grape pomace from winemaking in relation to ROS generation (Tsukada et al., 2016a,c) The extract was prepared as in the following way. Fruitage (including the peel and seeds) of the white wine grape variety Niagara harvested at Hokkaido in Japan was crushed and pressed to obtain a juice in the vinification process of white wine. Then the remnant of crushed and pressed grapes (grape pomace) was freeze-dried. Three times the volume of pure water (at the ratio of 3 mL pure water per 1 g powder) was added to the dried residue powder, and the resultant mixture was agitated at 150 rpm overnight at room temperature. The upper layer was taken and centrifuged at 3,000 rpm for 20 min to obtain a supernatant. Filtrate through membrane filtration (φ0.22 μm) was adjusted to contain designated concentrations of total polyphenols, and was used for the assays. The resultant aqueous extract solution was termed as grape pomace extract (GPE) hereafter.

Qualitative and quantitative analyses of ·OH generated by photoirradiation of GPE were performed using an electron spin resonance (ESR) spin trapping technique with 5,5-dimethyl-1-pyrroline *N*-oxide (DMPO) as a spin trap. In the assay, 300 mM DMPO was used because it was reported that 300 mM DMPO is optimal to trap μM level of ·OH sufficiently (Nakamura et al., 2010). The sample (GPE) was irradiated with a light emitting diode (LED) with a wavelength of 400 nm for 0, 10, 20, and 60 s. After irradiation, determination of DMPO-OH (a spin adduct of DMPO and ·OH) was carried out with 4-hydroxy-2,2,6,6-tetramethylpiperidine *N*-oxyl (TEMPOL) as a standard to calculate the concentration of DMPO-OH. To confirm if DMPO-OH was derived from the reaction between free ·OH and DMPO, dimethyl sulfoxide (DMSO), a representative ·OH scavenger was added to the reaction mixture, resulting in that DMPO-OH signal decreased, and a signal for DMPO-CH_3_ was observed (Figure 1). The yields of DMPO-OH after LED-light irradiation of GPE increased linearly with time up to 20 s, and the yield kept similar level after that. When the photoirradiated GPE for 20 min without DMPO was furthered irradiated with LED light for 10 s in the presence of DMPO, the yield of DMPO-OH was similar to that after 10 s irradiation of GPE without prior irradiation. That is, even after 20 min photoirradiation, newly added DMPO trapped ·OH indicating that ·OH was generated during photoirradiation for 20 min or longer. Likewise, further study revealed that ·OH was continuously generated at least up to a couple of hours, even though DMPO-OH level reached a plateau in a short period of time. Besides ·OH, H_2_O_2_ yield determined by a colorimetric method based on the peroxide-mediated oxidation of Fe^2+^ followed by the reaction of Fe^3+^ with xylenol orange also increased with irradiation time, confirming the idea that photooxidation of polyphenols contained in GPE resulted in H_2_O_2_ generation and subsequent ·OH formation via homolytic cleavage of H_2_O_2_ (Figure 1). When ·OH yield of photoirradiation of GPE was compared to that of commercially available grape seed extract (GSE, Leucoselect®, Indena S.p.A., Milan, Italy) and (+)-catechin, GPE was more potent than GSE and (+)-catechin.

**Figure 2:**
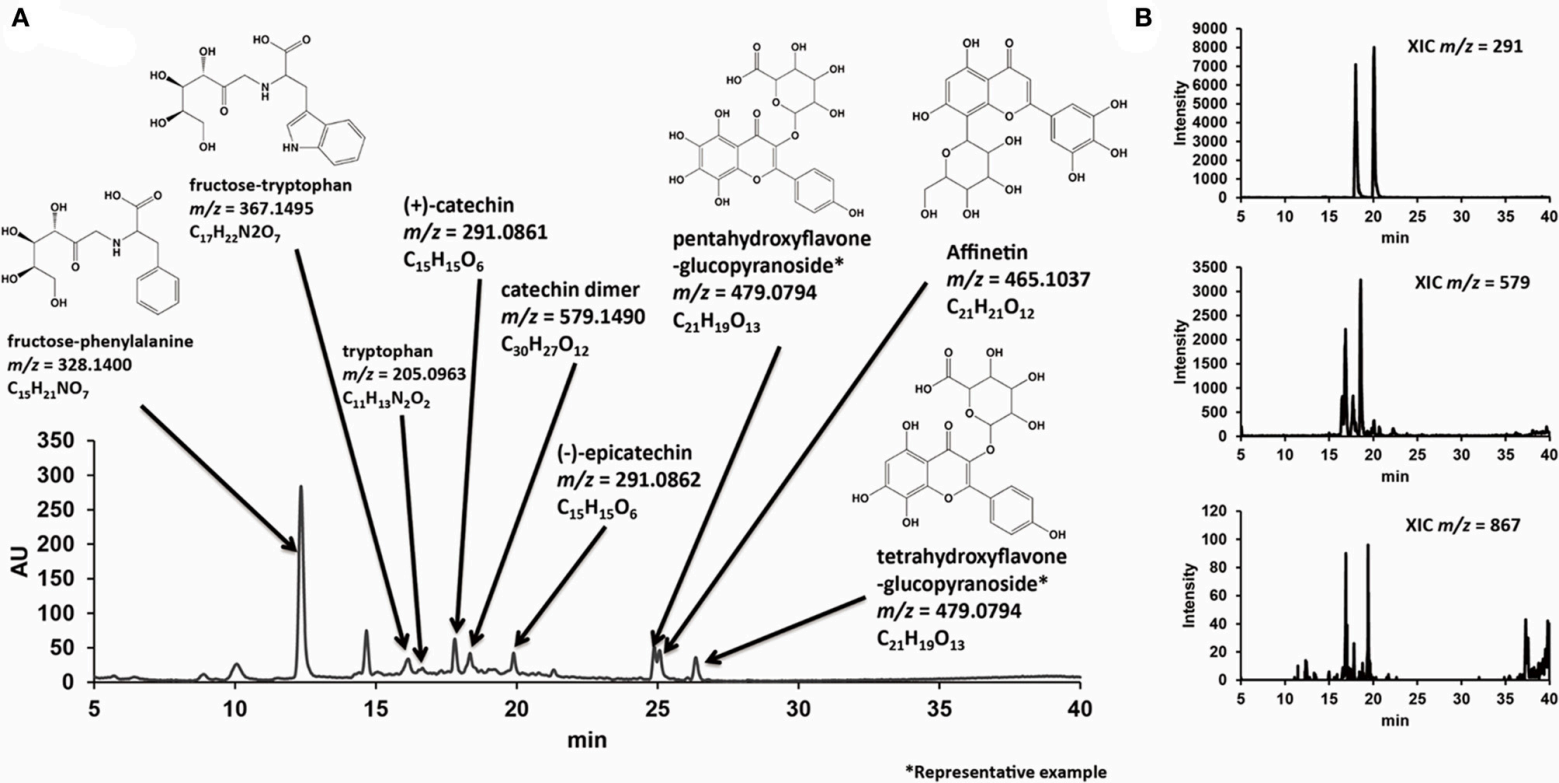
Representative LC chromatogram of GPE with estimated chemical structural formulas determined by MS analysis **(A)**, and extracted-ion chromatograms (XICs) with m/z corresponding to catechin monomers, dimers, and trimmers **(B)**. Reproduced from Tsukada et al. (2016a) with permission.

Regarding polyphenols in GPE, liquid chromatography-electrospray ionization-mass spectrometry (LC-ESI-MS) analysis showed that polyphenolic compounds including catechin monomers, dimers, trimers, and polyphenolic glucosides were contained (Figure 2).

## Bactericidal activity of photoirradiation of GPE

Potent bactericidal activity of photoirradiation of GPE was demonstrated against *Staphylococcus aureus* (Tsukada et al., 2016a,c). When *S. aureus* suspended in GPE was irradiated with LED light at 400 nm for 20 min, a 5-log or greater reduction in viable bacterial cells was achieved. Once ·OH scavengers were added to the suspension of *S. aureus*, the bactericidal effect of the photoirradiated GPE was prominently attenuated, revealing that the bactericidal activity of photoirradiation of GPE was mainly attributable to ·OH. In a study where the effect of wavelengths of light (365, 385, 400, and 465 nm with irradiance of 715 mW/cm^2^) was examined, absorption of light by GPE increased as the wavelength was shorter. Accordingly, when the extract was irradiated with the light at the four wavelengths, the shorter the wavelength was, the more H_2_O_2_ was yielded. In addition, it was revealed that H_2_O_2_ yield and bactericidal activity against *S. aureus* under LED-light irradiation increased inversely with the wavelength. Thus, bactericidal activity of photoirradiation of GPE would be augmented by controlling wavelength of light, which could be an advantageous point. Furthermore, when bactericidal activity of photoirradiation of GPE was compared to that of GSE and (+)-catechin, GPE was more potent than GSE and (+)-catechin, which also could give superiority to GPE.

Besides GPE, bactericidal effect of photoirradiation of aqueous extract from wine lees was examined (Tsukada et al., 2016b). Wine lees, also a major waste product of winemaking, was obtained from the same white wine grape variety Niagara as used in GPE, and its aqueous extract (termed as WLE) was similarly prepared as was the case with GPE. Bactericidal activity of photoirradiation of WLE against *S. aureus* was comparable to that of GPE, and also effective against *Pseudomonas aeruginosa*. However, unlike GPE, ·OH yield upon photoirradiation decreased with time. In that sense, we conclude that GPE is a better prooxidative disinfectant than WLE.

A recent study demonstrated that antimicrobial technique based on photooxidation of caffeic acid was highly effective against biofilm-forming *Streptococcus mutans*, the most common acidogenic bacterial species isolated from human cariogenic dental plaques, in relation to ·OH formation (Nakamura et al., 2017). Therefore, the authors concluded that photoirradiation of caffeic acid has the potential to be applied as an inexpensive antimicrobial therapy to prevent and treat dental caries. As such, one of the possible applications of photoirradiation of GPE would be oral infectious diseases (e.g. dental caries).

## Conclusion and future perspective

As total amount of grape pomace discharged from winemaking was globally estimated to be approximately 10 million tons, the pomace could be a valuable resource to be recycled. Besides antioxidative resources, one of its beneficial uses as a prooxidant is disinfection with following merits. Since the generation of ·OH from GPE is terminated by cessation of photoirradiation, the prooxidative action does not continue after the disinfection treatment. Even if excessive amount of ·OH is generated, the radicals cannot exist for long time because of its extremely short life time (Roots and Okada, 1975; Pryor, 1986). Therefore, the residual toxicity is practically negligible. Another beneficial point of utilizing ·OH is that the risk of inducing bacterial resistance is quite low because it interacts with several cell structures and different metabolic pathways in microbial cells, probably resulting in a lack of development of bacterial resistance (Ikai et al., 2013). Furthermore, GPE could be edible because it is derived from grape, so that GPE is a safe material to be handled. Accordingly, it is expected that this disinfection technique with photoirradiation of GPE is applicable to wide range fields. Since advanced oxidation process (AOP) can be mentioned as a similar technique, we propose that the technique utilizing photoirradiation of GPE could be applied to ways as in AOP. AOP is well known as a technique that combination of oxidant and ultraviolet (UV) can generate ·OH. One research group of University of Colorado has extensively studied on AOP application to frontier technologies for water processing and wastewater disinfection such as degradation of organic contaminant and microbial inactivation (Keen et al., 2012, 2016; Keen and Linden, 2013; Lester et al., 2016; Parker et al., 2017). Of their studies, they reported that combination of H_2_O_2_ and low dose UV inactivated adenovirus that is a pathogen resistant to UV disinfection (Bounty et al., 2012). They suggested that ·OH generated by this AOP damaged proteins that are not UV target, resulting in enhanced inactivation effect of UV. As such we also expect that the disinfection effect of photoirradiation of GPE could be augmented by changing extraction solvent to extract polyphenols more sufficiently, breed variety of grape to be richer in polyphenols, and wavelength of light to generate ·OH more effectively, leading to application to environmental cleanup as reported by the studies on AOP.

The other similar disinfection technique utilizing ·OH is photolysis of H_2_O_2_ originally designed for the treatment of periodontitis. Irradiation of blue visible light to H_2_O_2_ generate ·OH that in turn exert not only *in vitro* bactericidal activity but disinfection effect on a superficial bacterial infection model in rats (Ikai et al., 2010; Hayashi et al., 2012). In addition, *in vivo* studies showed that the acute locally injurious property of the disinfection technique is considerably low (Yamada et al., 2012; Sato et al., 2016). Thus, further studies should be conducted in the near future to examine if the disinfection technique with photoirradiation of GPE could be applied to the treatment of superficial infection including oral infectious diseases as describe above.

## Author contributions

MaT and YN wrote the manuscript, and YN and MiT edited the manuscript. All authors approved the final version of the manuscript.

### Conflict of interest statement

The authors declare that the research was conducted in the absence of any commercial or financial relationships that could be construed as a potential conflict of interest.
